# Expression Profiling of Mitogen-Activated Protein Kinase Genes Reveals Their Evolutionary and Functional Diversity in Different Rubber Tree (*Hevea* *brasiliensis*) Cultivars

**DOI:** 10.3390/genes8100261

**Published:** 2017-10-06

**Authors:** Xiang Jin, Liping Zhu, Qi Yao, Xueru Meng, Guohua Ding, Dan Wang, Quanliang Xie, Zheng Tong, Chengcheng Tao, Li Yu, Hongbin Li, Xuchu Wang

**Affiliations:** 1 Institute of Tropical Biosciences and Biotechnology, Chinese Academy of Tropical Agricultural Sciences, Haikou 571101, China; jinxiang@itbb.org.cn (X.J.); zhuliping0903@163.com (L.Z.); yaoqi7@outlook.com (Q.Y.); xuerumeng1203@126.com (X.M.); dingguohuasw@163.com (G.D.); wangdan@itbb.org.cn (D.W.); tongzheng@itbb.org.cn (Z.T.); 2 College of Life Sciences, Key Laboratory of Agrobiotechnology, Shihezi University, Shihezi 832003, China; xiequanliang001@163.com (Q.X.); taocc_124@163.com (C.T.); yulixjnu@163.com (L.Y.)

**Keywords:** *Hevea brasiliensis*, mitogen-activated protein kinase, natural rubber biosynthesis, gene family evolution, functional diversity, abiotic stress

## Abstract

Rubber tree (*Hevea* *brasiliensis*) is the only commercially cultivated plant for producing natural rubber, one of the most essential industrial raw materials. Knowledge of the evolutionary and functional characteristics of kinases in *H. brasiliensis* is limited because of the long growth period and lack of well annotated genome information. Here, we reported mitogen-activated protein kinases in *H. brasiliensis* (HbMPKs) by manually checking and correcting the rubber tree genome. Of the 20 identified HbMPKs, four members were validated by proteomic data. Protein motif and phylogenetic analyses classified these members into four known groups comprising Thr-Glu-Tyr (TEY) and Thr-Asp-Tyr (TDY) domains, respectively. Evolutionary and syntenic analyses suggested four duplication events: *HbMPK3/HbMPK6*, *HbMPK8/HbMPK9/HbMPK15*, *HbMPK10/HbMPK12* and *HbMPK11/HbMPK16/HbMPK19*. Expression profiling of the identified *HbMPK*s in roots, stems, leaves and latex obtained from three cultivars with different latex yield ability revealed tissue- and variety-expression specificity of *HbMPK* paralogues. Gene expression patterns under osmotic, oxidative, salt and cold stresses, combined with *cis*-element distribution analyses, indicated different regulation patterns of *HbMPK* paralogues. Further, *Ka*/*Ks* and Tajima analyses suggested an accelerated evolutionary rate in paralogues *HbMPK10/12*. These results revealed *HbMPKs* have diverse functions in natural rubber biosynthesis, and highlighted the potential possibility of using MPKs to improve stress tolerance in future rubber tree breeding.

## 1. Introduction

The rubber tree (*Hevea brasiliensis*) is the most important commercially cultivated natural rubber-bearing plant. It is a member of the spurge family (*Euphorbiaceae*) and is closely related to cassava (*Manihot esculenta*) and castor bean (*Ricinus communis*). Natural rubber (*cis*-1,4-polyisoprene) is strategically important, and no other synthetic alternatives possess the exact same physicochemical properties. Therefore, rubber tree has substantial economic value as the only commercially cultivated source of natural rubber [1]. Although natural rubber has a long use history for industrial materials, molecular biological studies on *H. brasiliensis* (termed as *Hevea* hereafter) remain limited, because of its narrow and restricted cultivation region (only in tropical and partial subtropical area) and the long growth time (at least 7 years) before it becomes available for industrial use [2]. The first draft rubber tree genome was released in 2013 [3], but the sequence coverage is very low, and the genome assembly is also limited. Recently, a better draft rubber tree genome has been released [4]. The estimated size is 1.46 GB, and the best draft genome published up-to-date assembled 1.36 GB (93.8% of genome), including 7453 scaffolds (weighted median scaffolds size N50 = 1.28 MB), which contain 43,792 predicted protein coding genes. These released rubber tree genomes have greatly improved the investigations of gene and protein functions in rubber tree [3,4,5]. Mitogen-activated protein kinase (MPK) cascades are evolutionarily conserved signal transduction modules that contain three kinase models, including mitogen-activated protein kinase kinase kinases (MPKKKs), mitogen-activated protein kinase kinases (MPKKs), and MPKs [6,7]. Signalling *via* MPK cascades in higher plants is important with respect to various plant development processes, including gene expression regulation, biotic and abiotic stress tolerance, and phytohormone responses [8,9,10]. As the last component of MPKKK-MPKK-MPK cascades, MPKs constitute a specific family of Ser/Thr protein kinases, and play crucial roles in transduction of extracellular stimuli in higher plants by phosphorylating various downstream targets [11,12,13]. MPK family members contain either a Thr-Glu-Tyr (TEY) or a Thr-Asp-Tyr (TDY) phosphorylation motif within their active site; these motifs can be classified into four major groups (groups A, B and C for the TEY type, and group D for the TDY type) [14].

Twenty and seventeen MPKs have been identified in the model plants *Arabidopsis thaliana* [15] and *Oryza sativa* [16], respectively. Furthermore, a total of 17, 12, 15, 17, 38, 21, 26, and 20 MPKs have been identified in maize [17], grape [18], wheat [19], tobacco [20], soybean [21], poplar [22], apple [23], and cassava (*M. esculenta*) [24], respectively. In general, fewer MPK family members are found in monocotyledons compared with dicotyledons, which indicates that gene family expansion events mainly occurred in dicots during the divergence of monocots and dicots.

MPK members in groups A and B play important roles in tolerance to abiotic stresses, such as cold, drought and salt. In *Arabidopsis*, *AtMPK6* is required for cold stress resistance owing to its phosphorylation of the transcriptional repressor a MYB DNA-binding domain containing transcription factor (MYB15) [25]. Gawroriski et al. reported that *mpk4* mutant caused imbalance in the homeostasis of reactive oxygen species (ROS) in *Arabidopsis* [26]. Moreover, *AtMPK3* and *AtMPK6*were reported to play vertical roles in plant innate immunity against parasitic nematodes [27]. *OsMPK5* can improve rice salt tolerance [28], and *OsMPK33* is involved in drought stress tolerance [29]. Nevertheless, knowledge of the MPK gene family in rubber-bearing plants remains limited.

An important technique for increasing natural rubber yield under abiotic and biotic stresses involves mining key genes for stress tolerance improvement in rubber tree. Here, we manually reviewed the genome database and identified 20 MPKs in *Hevea*, four members of which were detected from our proteomic data. Sequence and evolutionary characteristics were also investigated. The expression profiles of *HbMPKs* in different tissues obtained from three cultivated varieties with different rubber yield ability were analysed using quantitative real-time PCR (qRT-PCR) and semi-quantitative PCR. The responses of *HbMPKs* to various abiotic stresses in one main cultivated variety were also determined. The different expression patterns of duplicated paralogues were observed and interpreted by the evolutionary rate and *cis*-element analyses of *HbMPKs*. This study provides an overview of MPKs in the most important rubber-bearing *Hevea* plant, and reveals the potential possibility of exploiting MPKs to improve stress tolerance in future rubber tree breeding.

## 2. Materials and Methods

### 2.1. Identification and Genomic Location of HbMPK Genes

Previously reported MPKs of *Arabidopsis* [15] and rice [16] were retrieved and subjected to a Basic Local Alignment Search Tool for protein query (BLASTP) against the *Hevea* genome database, in order to comprehensively annotate MPKs [4]. Candidate *HbMPK* gene sequences were then submitted to InterProScan [30] to assess the MPK conserved motif (IPR003527) and protein kinase domain (IPR000719). A careful manual review of the resultant *HbMPK* candidate genes was performed to correct any potential mistakes in the genome database.

The genomic locations of these identified *HbMPK* genes were further determined based on the results of a BLAST nucleotide (BLASTN) query against the *Hevea* genome sequence. MapInspect software was used to draw the locations of the *HbMPK* genes.

### 2.2. Phylogenetic Tree, Motif Distribution and Alignment of the Mitogen-Activated Protein Kinase Family

The amino acids that compose MPKs from *Hevea*, two model plants *Arabidopsis* and rice, and a closely related cassava species [31] were identified, after which a phylogenetic tree was constructed by MEGA5.0 [32] using neighbour-joining (NJ) method. Bootstrap tests were performed and consisted of 1000 replicates.

Multiple sequence alignment was performed using ClustalW [33]. The molecular weight and isoelectric points of predicted HbMPK proteins were estimated using the ExPASy proteomics server [34]. The subcellular localization of HbMPKs was predicted using Softberry [35]. Conserved motifs of the HbMPK proteins were analysed using the Multiple Expectation Maximization for Motif Elicitation (MEME) program [36].

### 2.3. Gene Structure, Duplication Events and Syntenic Analyses

Gene Structure Display Server (GSDS) software [37] was used to analyse the exon-intron distribution based on the comparison of the open reading frame (ORF) sequence and genomic coding region of each *HbMPK* gene.

Gene duplication events were determined by multiple sequence alignment and collinear analyses. Homologous *HbMPK* genes were considered as paralogues only when their nucleotide identities were >90% [38,39]. The *Ka* (nonsynonymous substitution rate) and *Ks* (synonymous substitution rate) were calculated using DnaSP 5.0 software [40]. The *Ka/Ks* ratios for the *HbMPK* genes were calculated to assess the selection pressure on duplicated genes; a *Ka/Ks* ratio >1, <1, or =1 indicates positive, negative, or neutral evolution, respectively [41]. Tajima relative rate tests [42] were performed using the amino acid sequences of the duplicated *HbMPK* pairs. The syntenic relationships of paralogues and/or orthologues among *Arabidopsis*, cassava, and rubber tree were analysed using the Circos program [43].

### 2.4. Plant Materials and Abiotic Stress Treatments

Three rubber tree cultivated varieties, Reyan8-79 (high yielding), Reyan7-33-97 (medium yielding), and PR107 (low yielding), were used in this study. Different tissues (roots, stems, leaves and latex) were collected from the ten-year-old mature trees, and immediately frozen in liquid nitrogen. One-year-old Reyan7-33-97 seedlings were used for abiotic stress treatments. For osmotic and salt stress treatments, the seedlings were irrigated with 20% polyethylene glycol (PEG) 6000 and 1 M NaCl, respectively. For oxidative stress, the leaves were sprayed with 2% (*v*/*v*) H_2_O_2_. For cold stress, the seedlings were placed in a growth chamber at 4°C. For RNA extraction, the leaves were collected at 3 h, 12 h, and 24 h after stress treatment. Untreated rubber tree leaves were used as 0 h control.

### 2.5. RNA Extraction and Expression Profiling of HbMPKs

The total RNA from different tissues (roots, stems, leaves and latex) of three cultivated varieties and the leaves of Reyan7-33-97 following abiotic stress treatments were extracted using an RNAprep Pure Plant Kit (Tiangen, Beijing, China). First-strand complementary DNA (cDNA) was generated using a reverse transcription system (Takara, Kusatsu, Japan) in accordance with the manufacturer’s instruction.

The qRT-PCR was conducted using the gene specific primers listed in Supplementary Table S1. The reactions were performed using a Stratagene Mx3005P Real-Time Thermal Cycler (Agilent, Santa Clara, CA, USA) and SYBR Green Master Mix Reagent (Takara, Kusatsu, Japan), in accordance with previously described PCR conditions [44]. The specificity of the reaction was verified by melting curve analysis. The 2^−ΔΔCT^ method was used to calculate the relative expression level of the target genes. The *HbActin* (GenBank Acc. HQ260674.1) served as a reference gene. Three independent biological replications were performed for each gene. Heat maps were generated using Multi Experiment Viewer (MeV) software as described [45]. Semi-quantitative PCR assays were performed using the same cDNA templates and primers used for qRT-PCR. The template concentrates were normalised using the *HbActin* gene as a reference. The expression levels of each *HbMPK* gene were visualised using agarose gel electrophoresis of the corresponding PCR products.

### 2.6. Cis-Element Distributions in HbMPK Promoter Regions

To investigate the *cis-*regulatory elements in the promotor regions of *HbMPKs*, a 1500 bp region of the genomic sequence up stream of the start codon of each gene was retrieved from the *Hevea* genome database. These sequences were then submitted to online PlantCARE software [46] in order to predict putative *cis-*elements.

## 3. Results

### 3.1. Identification and Characterization of MPK Members in Hevea

A total of 45 MPK candidates were identified by submitting the *A. thaliana* MPK (AtMPK) and *O. sativa* MPK (OsMPK) sequences to BLASTP queries against the *Hevea* genome database. Furthermore, 20 candidates that had both a protein kinase domain and a conserved MPK active site were determined to be HbMPKs after sequence analysis (Table 1). The 20 putative HbMPKs were carefully reviewed, after which the coding sequences (CDS) of *HbMPK15* (scaffold0949_294805) were manually corrected by investigating the downstream genomic sequences. The ORF lengths of the 20 *HbMPKs* ranged from 894 bp (*HbMPK17*) to 1848 bp (*HbMPK12*) and their encoded proteins consisting of 298 to 616 amino acids. The calculated molecular weights of these proteins ranged from 34.85 to 69.95 kDa, and their isoelectric points ranged from 4.81 to 9.28. These proteins were predicted to localise in the nucleus (16 members) or cytoplasm (4 members).

Previously obtained isobaric tag for relative and absolute quantitation (iTRAQ) based proteomic data of the Reyan7-33-97 rubber tree latex [47] were submitted to the *Hevea* genome database to determine the expression patterns of HbMPK proteins. As a result, 4 out of the 20 HbMPKs were validated by high-throughput tandem mass spectrometry (MS/MS) data using a threshold of 95% confident peptides ≥2; two of them are induced by ethylene stimulation (Table 2). 

**Figure 1:**
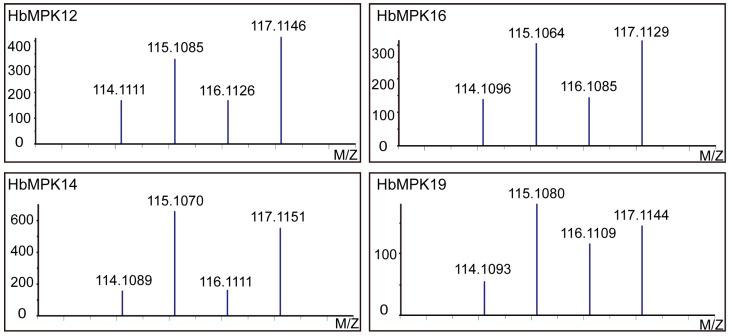
Proteomic validation of *Hevea brasiliensis* mitogen-activated protein kinases (HbMPK) expression patterns in rubber tree latex before and after ethephon treatment. Representative mass spectra indicate the signal intensities of isobaric tags for the validated MPKs. The spectra correspond to the peptides of “APELCGSFFSK”, “DYVHQLR”, “DLKPSNLLLNANCDLK” and “SFDFEQNALTEEQMK” for HbMPK12, HbMPK14, HbMPK16 and HbMPK19, respectively.

The raw spectra of isobaric tag reporters (Figure 1) and identified peptides that mapped to the HbMPKs are provided (Supplementary Figure S1). These data validated the above 20 putative HbMPKs at the proteomic level.

**Figure 2:**
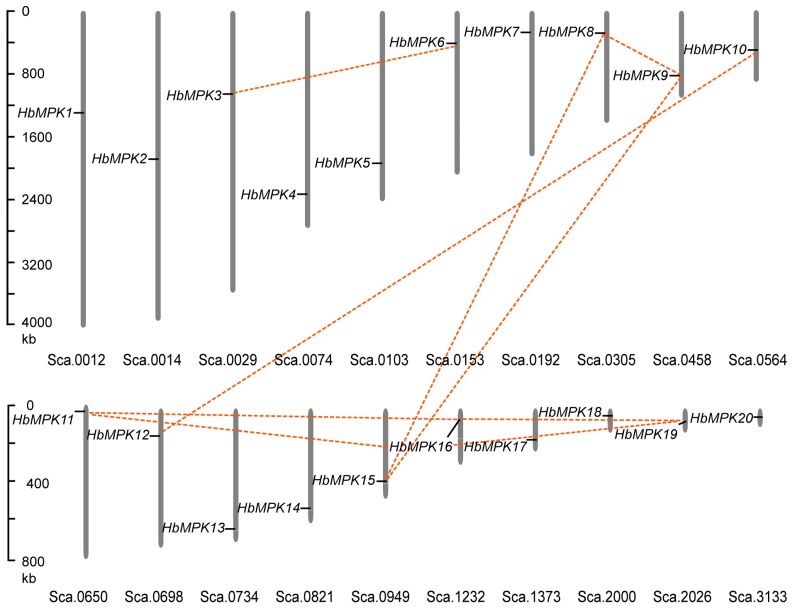
Scaffold distribution of *MPK* genes in *Hevea*. *HbMPK* family members were distributed onto 20 individual scaffolds. Scaffolds are represented by vertical columns, and scaffold numbers are shown at the bottom. Scale bars are on the left. Red lines connect paralogues.

Genomic location analysis showed that *HbMPK* family members are distributed onto 20 individual scaffolds (Figure 2). The rubber tree was considered to have undergone two rounds of genome duplication events [4]. The expansion of the *MPK* gene family in *Hevea* was further investigated by analysing duplication events in *MPK* genes. Four duplication events involving 10 paralogues were identified (*HbMPK3/HbMPK6*, *HbMPK8/HbMPK9/HbMPK15*, *HbMPK10/HbMPK12*, and *HbMPK11/HbMPK16/HbMPK19*), which indicates that segmental duplication events play significant roles in *MPK* gene expansion in the *Hevea* genome.

### 3.2. Multiple Sequence Alignment and Motif Distribution of HbMPKs

**Figure 3:**
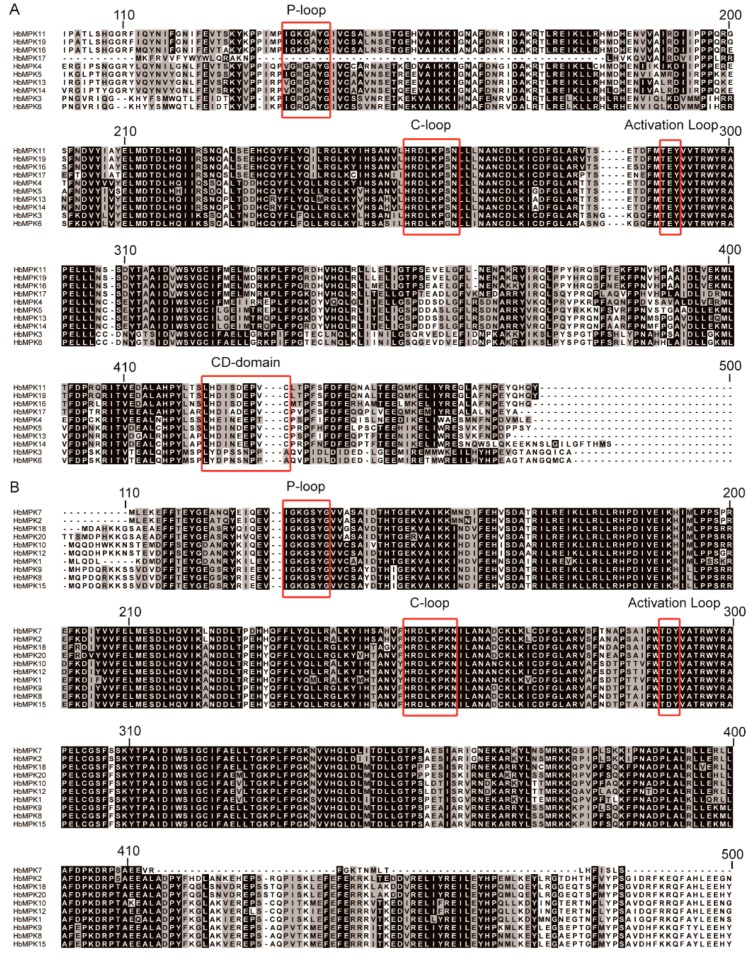
Multiple sequence alignment of the MPK proteins in *Hevea*. Conserved regions are shadowed. (**A**) Ten HbMPK members that contain a Thr-Glu-Tyr (TEY) activation site and a CD-domain; (**B**) Ten HbMPK members that contain a Thr-Asp-Tyr (TDY) active site. The alignment regions range from amino acids 100 to 500; each line consists of 100 amino acids. The four conserved domains (P-loop, C-loop, activation loop and CD-domain) are marked in red boxes.

To investigate the sequence conservation of HbMPK proteins, multiple sequence alignment and conserved motif distribution analyses were performed. The results of the multiple sequence alignment showed that all identified HbMPKs contain the previously reported MPK-specific conserved domains: an N-terminal ATP-binding domain (P-loop), a phosphorylation catalytic loop (C-loop), and an activation loop (TEY or TDY). Notably, the HbMPKs that contained the TEY-type (Figure 3A) activation loop also have an additional evolutionarily conserved C-terminal common docking domain (CD-domain), whereas the TDY-type (Figure 3B) subfamily proteins do not, indicating a different evolutionary origin and functional diversity among these proteins.

**Figure 4:**
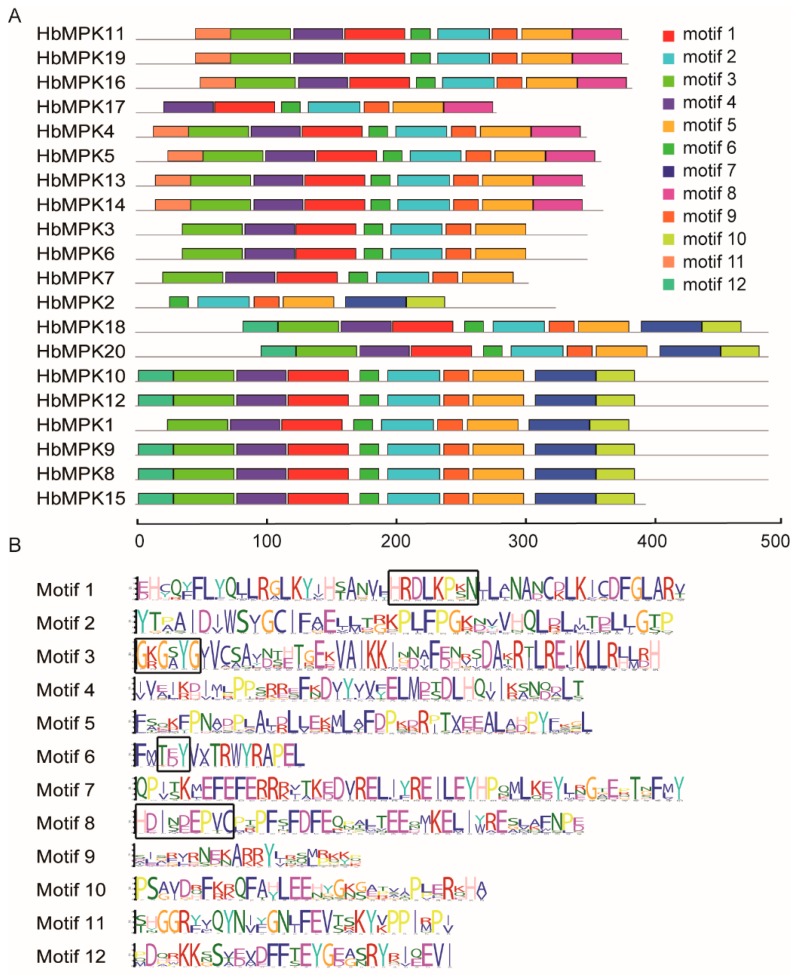
Motif analysis of the MPK family members in *Hevea*. (**A**) Motif distributions for 20 HbMPKs. Rectangles with different colour represent 12 recognised conserved motifs; (**B**) Detailed information (amino acid sequence) of the 12 motifs. The heights of each letter represent the frequency of amino acids at that position, and the black boxes indicate the key sites of the four conserved motifs.

Furthermore, 12 conserved motifs were recognised by the MEME program [36]. The distributions of the 12 conserved motifs for all 20 HbMPKs were highlighted (Figure 4A). The MPKs were phylogenetically ordered to better understand their evolutionary relationships. Motifs 2, 5, 6 and 9 are conserved in each HbMPK and are organised in the exact same order. Motif 8 is specific to the MPKs of groups A and B, which have the conserved CD-domain. The conserved amino acid sequence information of these 12 motifs was also shown (Figure 4B). Motifs 1, 3, 6, and 8 correspond to the conserved C-loop, P-loop, activation loop, and CD-domain, and were identified in the multiple sequence alignment (black boxes in Figure 4B).

### 3.3. Evolution and Exon-Intron Organization of HbMPKs

**Figure 5:**
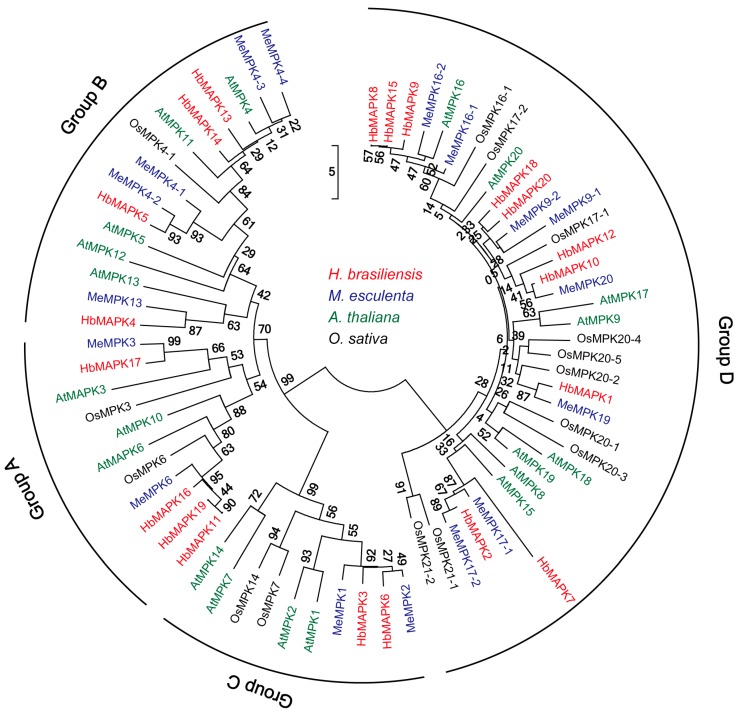
Phylogenetic tree of the MPK family in *Arabidopsis thaliana*, *Oryza sativa*, *Manihot esculenta*, and *Hevea brasiliensis*. The phylogenetic tree was constructed by ClustalX 2.0 and MEGA5.0 software using the neighbour-joining (NJ) method and 1000 bootstrap replicates. The names of the MPKs from the four species are shown in different colours. Four distinct clades (groups A–D) are highlighted. The vertical bar indicates evolutionary distance.

To investigate the evolutionary relationships between HbMPKs and the MPKs from other plants, a phylogenetic tree was constructed using the protein sequences of 17 MPKs from the monocot model plant rice (*O. sativa*), 20 MPKs from the dicot model plant *A. thaliana*, 16 MPKs from the rubber tree relative cassava (*M. esculenta*), and 20 from *Hevea* in this study. These MPKs were divided into four groups according to phylogenetic analysis: TEY-type MPKs compose groups A, B and C, and TDY-type MPKs compose group D (Figure 5). No species-specific clades were identified; however, groups A and B contain many more dicot MPK members than monocot MPK members. This finding indicates that duplication events may have occurred after the divergence of monocots and dicots. At the same time, group D contains 9 OsMPKs, 6 AtMPKs, 6 *M. esculenta* MPKs (MeMPKs), and 8 HbMPKs, which suggests multiple evolutionary and functional diversities for these MPK members. An *A. thaliana*-specific duplication event was identified in group C, and two potential *Hevea*-specific duplication events were identified in groups B (HbMPK11, 16 and 19) and D (HbMPK8, 9 and 15) by phylogenetic analysis (Figure 5).

**Figure 6:**
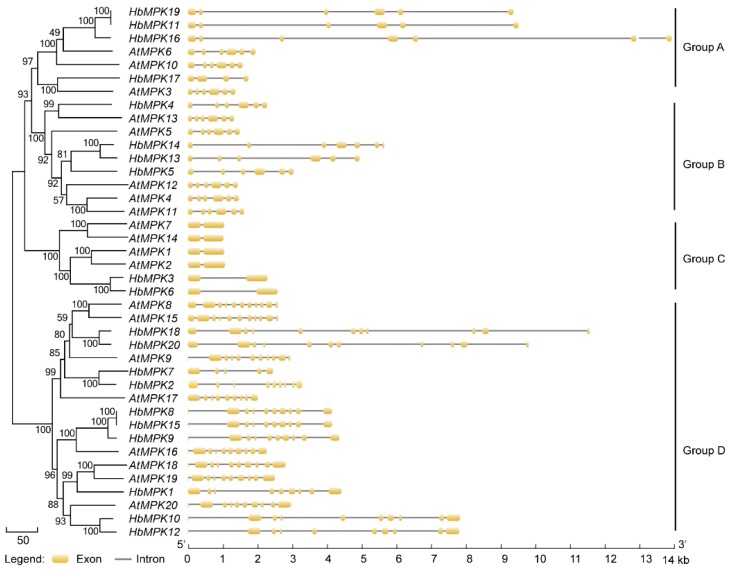
Phylogenetic and exon-intron structure analyses of *MPK* genes in *Arabidopsis* (*At*) and *Hevea* (*Hb*). The cladogram (left) was constructed using MEGA5.0 as described above. The exon-intron distribution (right) was analysed using GSDS software. Introns and exons are represented by lines and yellow boxes. The scale bar for genomic length is indicated at the bottom.

Gene structure divergence plays important roles in the evolution of gene families and can be used to assess phylogenetic relationships [48]. Analyses of the phylogenetic relationships and exon-intron distributions of both *AtMPKs* and *HbMPKs* revealed that most members in the same group shared very similar exon-intron structures (Figure 6). This similarity provides evidence of *MPK* classification and evolutionary relationships between *Arabidopsis* and *Hevea*. Group A and B *MPKs* contain 4–7 exons of similar length, and 3–6 introns of variable size, whereas group C members have 2 parallel exons and only 1 intron. Group D shows a complex distribution of exons and introns. We also observed that the gene structures are conserved between *HbMPK* and *AtMPK* homologues. Additionally, similar gene structures were observed not only among paralogues, but also among homologues, which indicates the possible origin of these MPKs and evolutionary duplication events (*AtMPK6* compared with *HbMPK11*/*HbMPK16*/*HbMPK19*, *AtMPK16* compared with *HbMPK8/HbMPK9/HbMPK15*, and *AtMPK20* compared with *HbMPK10/HbMPK12*). Moreover, we determined the *Hevea*-specific duplication relationships in the abovementioned paralogues by comparing the gene structure of *MPKs* to those of the related spurge family plant *M. esculenta*, and the result indicates that these duplicated *MPKs* are potentially involved in rubber tree-specific tissues and development processes (Supplementary Figure S2). However, the last duplication events, involving *HbMPK3/HbMPK6*, were observed in all of the investigated plant species investigated in this study (*AtMPK1/AtMPK2* and *MeMPK1/MeMPK2*). We are interested in the potential functional diversity of *Hevea*-specific duplicated MPKs.

**Figure 7:**
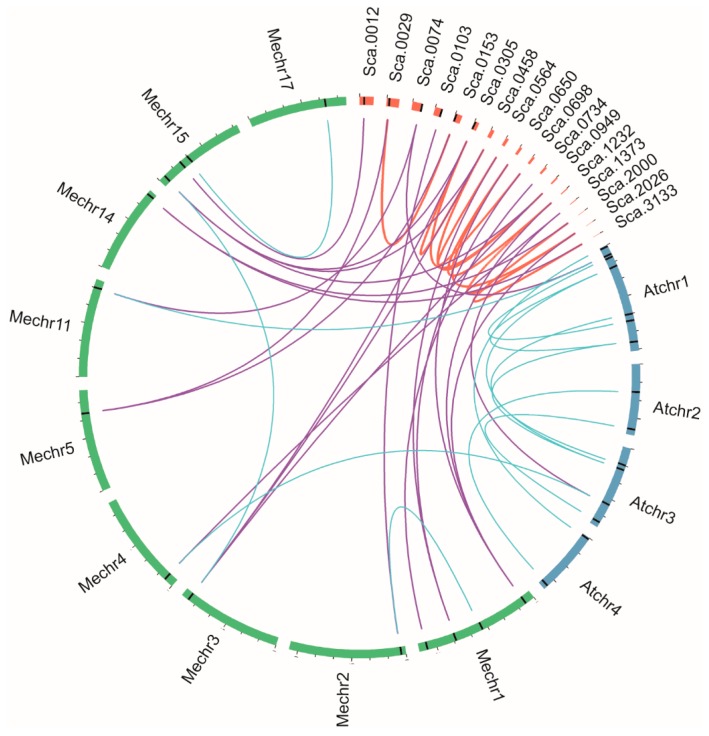
Segmental duplication of *HbMPK* genes and syntenic analysis of *Hevea*, *M. esculenta*, and *A. thaliana* MPKs. Chromosomes and scaffolds are shown in different colour in circular form. The positions of the MPK genes are marked by black lines on the circle. Duplicated MPK pairs in *Hevea* are connected by red lines. Syntenic relationships between *Hevea* and the other two species are connected by purple lines. Blue lines indicate MPK pairs between or inside *M. esculenta* and *A. thaliana*.

To further investigate the expansion of the *MPK* gene family in *Hevea*, syntenic analysis of *Hevea*, *M. esculenta*, and *A. thaliana MPKs* was performed. The results were visualised using Circos software. We identified eight segmental duplication pairs in *Hevea MPKs*, but no tandem duplication events were detected, as our results were partly limited by the lack of chromosomal assembly information for rubber tree genome (Figure 7). Again, the duplication events described above were determined to be syntenic genes: *HbMPK3/HbMPK6*–*MeMPK2*, *HbMPK10/HbMPK12*–*MeMPK20*, *HbMPK11/HbMPK16/HbMPK19*–*MeMPK6*, and *HbMPK8/HbMPK9/HbMPK15*–*MeMPK16-2*.

Modes of evolutionary selection can be estimated by the *Ka/Ks* ratio [41]. A *Ka*/*Ks* ratio > 1 indicates a positive selection, a *Ka*/*Ks* ratio < 1 indicates a purifying selection, and a *Ka*/*Ks* ratio = 1 indicates a neutral selection. The *Ka*/*Ks* ratios of the duplicated *HbMPKs* indicated that they all were subjected to purifying selection (Table 3). Furthermore, Tajima relative rate tests were conducted to investigate whether the *HbMPK* duplicates evolved at an accelerated rate following the duplication events. A statistically significant increase in evolutionary rate occurred between the *HbMPK10/HbMPK12* duplicated pairs (Table 4), which indicates a potential functional divergence of these duplicated paralogues.

### 3.4. Expression Profiling of MPKs in Different Tissues from Three Cultivated Varieties of Rubber Tree

**Figure 8:**
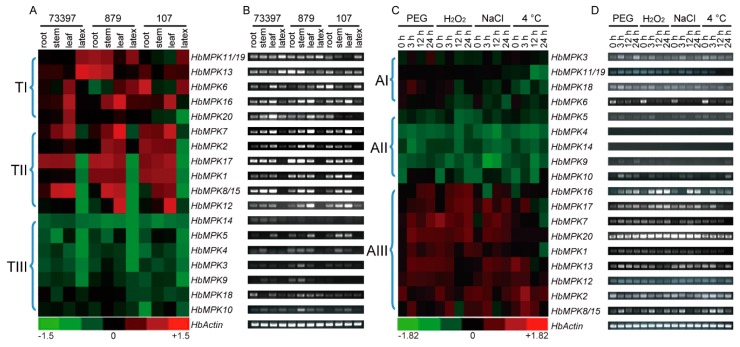
Expression profiles of *HbMPKs* in different tissues and stresses obtained from three cultivated varieties of rubber tree. Expression patterns of *HbMPKs* in different tissues from different cultivated varieties were determined using quantitative real-time PCR (qRT-PCR) (**A**), and semi-quantitative PCR (**B**). Moreover, heat map of qRT-PCR (**C**) and electrophoretogram of semi-quantitative PCR results (**D**) for 20 *HbMPKs* at 0 h, 3 h, 12 h, and 24 h under osmotic (polyethylene glycol, PEG), oxidative (H_2_O_2_), salt (1 M NaCl), and cold (4 °C) stresses are shown. The colour bar at the bottom indicates the log_2_ value of the expression fold change compared with that of the *HbActin* reference gene. The genes were ordered by the cluster of their expression patterns.

To gain insights on the tissue-specific expression patterns of the 20 *HbMPKs*, the expression patterns of *HbMPKs* were visualised as a heat map and separated into three clusters (Figure 8A). Members of cluster TI, including *HbMPK11/19* (these paralogues could not be distinguished by gene-specific primers), *HbMPK13*, *HbMPK6*, *HbMPK16*, and *HbMPK20*, showed the highest expression level in latex, and the members in cluster TI were distributed in all groups. Members of cluster TII showed universal expression patterns in all four tissues; the highest expression levels usually occurred in photosynthetic tissues. Most members in cluster TII were distributed in Group D, only HbMPK3 was distributed in Group A. The remaining *HbMPKs* were barely expressed. The cluster TIII did not have the members in Group A. The semi-quantitative PCR results were also shown by the electrophoresis analysis of the PCR products (Figure 8B). Notably, the MPKs in cluster TI (*HbMPK11/19*, *HbMPK13*, *HbMPK6*, *HbMPK16*, and *HbMPK20*), which are mostly expressed in the latex, also demonstrated different expression patterns among different rubber tree varieties, indicating that these genes might be involved in latex production processes in *Hevea*.

### 3.5. Expression Profiling of HbMPKs in Reyan7-33-97 under Different Abiotic Stresses

The genes of the MPK family are crucial abiotic stress responsive genes [49]. The expression patterns of *HbMPKs* under different abiotic stresses were analysed to further understand the functional diversity of *HbMPK* members. As shown in Figure 8, the 20 *HbMPKs* were grouped into three clusters according to their response to different abiotic stresses. The MPKs in cluster AI, especially *HbMPK6*, showed a weak or slightly reduced response to the abiotic stress treatments. Cluster AII included MPK family members that were barely expressed in leaves, either before or after abiotic stress. Cluster AIII contained the most *HbMPK* members, and showed different degrees of increased expression levels after abiotic stress (Figure 8C,D). Most members in cluster AIII were distributed in Group D, which means the members in Group D may play roles in diverse abiotic stresses. 

Regarding the different abiotic stresses, ten *MPKs* (*HbMPK1*, *3*, *7*, *8/15*, *10*, *13*, *16*, *17*, *18* and *20*) were up-regulated by osmotic stress (20% PEG 6000), whereas five (*HbMPK3*, *8/15*, *16*, *17* and *18*), seven (*HbMPK3*, *7*, *8/15*, *13*, *16*, *17* and *20*) and three (*HbMPK8/15*, *16* and *17*) members were up-regulated by H_2_O_2_, salt, and cold treatments, respectively. However, several *HbMPKs* were decreased following abiotic stresses: *HbMPK2* and *HbMPK6* under osmotic stress; *HbMPK2*, *HbMPK6* and *HbMPK13* under oxidative stress; *HbMPK2* and *HbMPK6* under salt stress; *HbMPK6* and *HbMPK13* under cold stress.

These expression pattern analyses of *HbMPKs* in different tissues and under different abiotic stresses suggested that *MPK* genes might have multiple functions in *Hevea* to adapt to various environmental changes (Figure 8). In addition, the duplicated paralogues showed very different expression profiles, indicating functional and evolutionary divergence emerged following the duplication events.

### 3.6. Analysis of cis-Elements in the Promotor Regions of MPKs in Hevea

**Figure 9:**
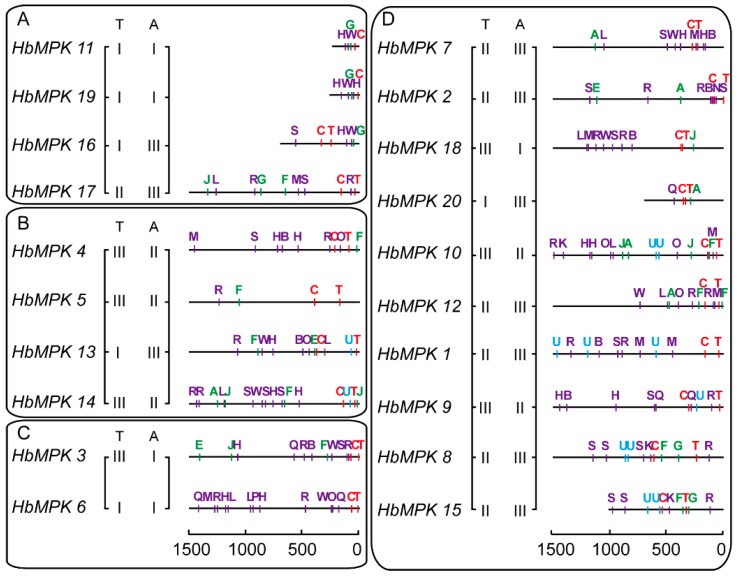
Distribution of *cis*-elements in the promoter regions of *HbMPK* genes. The *HbMPKs* were separated into four groups according to phylogenetic subfamily (groups A–D). The numerals in two parallel columns represent the cluster in the *HbMPKs* expression profiles in Figure 8. Coloured capital letters represent different *cis*-elements at corresponding positions. Red, green, purple, and blue letters represent the transcription initiation, phytohormone response, stress response, and high transcription classifications of *cis*-elements, respectively. Detailed information for each *cis*-element provided (Supplementary Table S2).

The distribution of *cis*-elements in the promoter regions of *HbMPKs* was further investigated to demonstrate the regulatory patterns of the *MPK* family members, especially duplicated paralogues (Figure 9). To facilitate this understanding, *cis*-elements were symbolised by capital letters in different colour; detailed classification and sequence information are listed (Supplementary Table S2). No significant positive correlations were observed between the distribution patterns of *cis*-elements and the expression patterns of *HbMPKs*, partly because of the lack of promoter sequence information for several genes. However, we did observe different *cis*-element organisational patterns in paralogues that exhibited different expression patterns in various tissues and/or under different abiotic stresses (*HbMPK3*–*HbMPK6*, *HbMPK10*–*HbMPK12*, and HbMPK11/19–HbMPK16). These results suggest the existence of different regulatory patterns and potential evolutionary fates in these paralogues.

## 4. Discussion

### 4.1. Identification and Characteristics of HbMPK Genes and TheirAssociated Proteins

MPKs in various higher plants, including *Arabidopsis*, rice, maize, tobacco, poplar, and cassava, etc., have been systematically investigated [15,16,17,18,19,20,21,22,23,24]. However, for the first time, we verified 20 *MPK* genes in the most important natural rubber-bearing plant. Rubber tree MPK family members share three conserved domains and could be separated into four known groups; similar results were observed in other plants. Three of these groups have a TEY-type activation loop, and 1 has a TDY-type activation loop [14]; the TEY-type MPKs also contain additional C-terminal CD-domains (Figure 3, Figure 4 and Figure 5). The genome of rubber tree is believed to have undergone two rounds of duplication events, resulting in similar numbers and characteristics of *HbMPKs* compared with those of *Arabidopsis* and *M. esculenta*.

To further verify the identified MPKs in rubber tree, previous iTRAQ data [47] obtained from rubber tree latex were introduced (Table 2; Figure 1). This is the first time that proteomic data were combined with genome-wide gene family studies for rubber-bearing plant research. The results support the gene family identification data and provide insights into the proteome patterns of MPK members in *Hevea*. Similar to genome research projects, proteomic data should be increasingly introduced in future gene-wide gene family studies. Although the proteomic data shown here did not provide the expression information about MPK proteins under abiotic stresses, we still believe that it is necessary to add proteomic evidence in the gene family investigations.

### 4.2. Evolutionary Divergence of Rubber Tree MPK Genes

The expansion of a gene family within a genome mainly occurs by three kinds of duplication events: tandem duplication of individual genes, segmental duplication of multiple genes, and background duplications that cannot easily be classified [50]. Given the limited chromosome assembly information of *Hevea*, the present study revealed that there is no or little contribution from tandem duplication to the expansion of the rubber tree *MPK* family (Figure 2 and Figure 7).

One important purpose of the evolutionary study of gene families is to determine whether there are species-specific family members. This information can aid in understanding the functional divergence of a specific gene family [51]. Although no rubber tree-specific groups were determined by phylogenetic, gene structure, or gene syntenic analyses of the *HbMPK* family, we identified rubber tree-specific duplication events (Figure 5, Figure 6 and Figure 7). Three *Hevea*-specific duplication events could be determined, but they were not observed for *Arabidopsis* or the spurge family plant cassava. These duplication events are *HbMPK11/HbMPK16/HbMPK19*–*AtMPK6*, *HbMPK8/HbMPK9/HbMPK15*–*AtMPK16*, and *HbMPK10/HbMPK12*–*AtMPK20*. The remaining paralogues demonstrate no species specificities, and *HbMPK3/HbMPK6* duplication was observed in all of the investigated plants. These results indicate that all these duplication events occurred at different time points of evolution *(*Figure 6 and Supplementary Figure S2).

Functional diversity caused by gene duplication might result in altered expression profiles and protein properties, and gene duplication is a major evolutionary driver for increasing the fitness of plants to new environment [52]. Furthermore, these paralogous pairs show tissue- and variety-specific expression patterns (Figure 8) as well as abiotic stress-specific patterns (Figure 8). One of the duplicated genes of three paralogous pairs shows global expression patterns in most tissues (*HbMPK6*, *HbMPK8/15*, *HbMPK12*, *HbMPK11/19*, and *HbMPK16*), whereas the other paralogue copies are barely expressed in root, stem, leaf, and latex tissues (*HbMPK3*, *HbMPK9*, and *HbMPK10*). Notably, the paralogues*HbMPK11/HbMPK16/HbMPK19* show universal expression patterns in different tissues (roots, stems, leaves, and latex) and under different abiotic stresses (osmotic, oxidative, salt and cold); however, *HbMPK11/19* are mainly expressed in rubber latex (Figure 8). These results suggest that *HbMPK16* might play similar role as its *AtMPK6* homologue in *Arabidopsis* (which is universally expressed in various tissues, and is strongly associated with numerous abiotic stresses) [53] and that *HbMPK11/19* somehow acquired new functions during the evolution of *Hevea*, and thus, are involved in nature rubber biosynthesis.

The selection pressure on *HbMPK* paralogues was estimated by calculating the ratio of *Ka*/*Ks* of rubber tree *MPK* paralogues. All *Ka*/*Ks* ratios were less than 0.1, which suggests that these paralogues were subjected to purifying selection, and mutations that alter amino acid sequences were not accepted during evolution (Table 3). These results are consistent with MPK family members playing very important roles during plant development, which requires highly conserved amino acid sequences to maintain protein functionality. Furthermore, the Tajima relative rates of *Hevea* gene pairs were calculated to evaluate the evolutionary rate between paralogues (Table 4). Notably, the duplicated gene pair *HbMPK10/HbMPK12* has a *p*-value of 0.00555, which means that one of the two duplicates has a significant accelerated evolutionary rate. After combining and analysing the different expression patterns between *HbMPK10* and *HbMPK12* both in various tissues and under abiotic stresses, we assumed that the two genes are undergoing an accelerated separation in *Euphorbiaceae* plants, which suggests that they potentially play specific roles in *Hevea*.

### 4.3. Expression Characteristics of HbMPKs in Various Tissues and Cultivated Varieties under Different Stresses

*MPK* genes in groups A and B are more like the mainly expressed *MPK*s [54]. We observed different expression patterns of *MPKs* in vegetative tissues (roots, stems, and leaves) and in specialised rubber tree tissue (latex); these *MPK*s mainly belong to groups A and B.

Five *MPK* genes (*HbMPK6*, *11/19*, *13*, *16* and *20*) were expressed in latex, according to the qRT-PCR and semi-quantitative PCR results (Figure 8). Notably, *HbMPK11/19*, *HbMPK13*, and *HbMPK6* showed significantly different expression levels across the high (Reyan8-79), medium (Reyan7-33-97), and low (PR107) yielding varieties of rubber tree. In particular, the expression levels of *HbMPK11/19* and *HbMPK13 in latex showed similar change patterns across the cultivated varieties,* whereas *HbMPK6* showed opposite patterns, which indicates that these genes may participate in the induction or repression of the biogenesis of natural rubber. The phosphorylation of functional proteins, including rubber elongation factor (REF), small rubber particle protein (SRPP), and osmotin, plays a very important role in natural rubber biosynthesis [55,56,57], and thus provides potential targets for *HbMPK6*, *HbMPK13*, and *HbMPK11/19*. In addition, two other *MPKs*, named *HbMPK20* and *HbMPK5*, have shown very different expression patterns across the three cultivated varieties, the reason for which remains unclear.

The majority of the *HbMPKs* can respond to various abiotic stresses in this study (Figure 8). The expression patterns under abiotic stress do not match the *cis*-element distributions in their promoter regions; however, the duplicated paralogues that have various *cis*-element distributions really exhibit completely different expression patterns both in different tissues and in different cultivated varieties under various abiotic stresses (Figure 8 and Figure 9). This phenomenon supports the hypothesis that gene loss occurs very rapidly following whole-genome duplication, and that functional divergence can explain why duplicated genes escape extinction [58].

## 5. Conclusions and Prospects

In summary, based on our proteomic data and the released *Hevea* genome, a total of 20 *Hevea* MPK family members were characterised. Phylogenetic and syntenic analyses determined that 10 of these *HbMPKs* are paralogues. Evolutionary analysis showed that all the paralogues were subjected to purifying selection, and that only one duplicated gene pair (*HbMPK10/HbMPK12*) has an accelerated evolutionary rate. Furthermore, expression profiling of the 20 *HbMPKs* in the roots, stems, leaves, and latex of three rubber tree cultivated varieties showed very different expression patterns among the paralogues. The paralogues of *HbMPK11/19* and *HbMPK16* showed very different expression levels in the latex of various rubber tree varieties, which indicates functional divergence between these gene pairs. Significantly different *cis*-element distributions were observed in the promoter regions of the duplicated paralogues. Our data provided the first systematic and evolutionary aspects of the MPK gene family of the rubber-bearing plant *Hevea*, and suggest that members of duplicated gene pairs, such as *HbMPK11/19* and *HbMPK13*, might play crucial roles in the biogenesis of natural rubber.

However, the limitation of transgenic technology in rubber tree and the lack of *Hevea* MPK-specific antibodies lead to a deficiency in functional validation in the present study. Moreover, additional investigations of several *HbMPK* paralogues (*HbMPK8/HbMPK9/HbMPK15* and *HbMPK10/HbMPK12*) that showed interesting expression patterns should be conducted in the near future.

## Figures and Tables

**Table 1:** Detailed information on putative *HbMPK* genes.

Gene	Scaffold Location ^a^	ORF (bp) ^b^	Predicted Protein ^c^			Subcellular Location ^d^
			Length (aa)	MW (kDa)	*p*I	
*HbMPK1*	scaffold0012 (−): 1243414–1248441	1812	604	68.36	9.13	Cytoplasmic
*HbMPK2*	scaffold0014 (−): 1804362–1809757	1035	345	38.98	8.40	Cytoplasmic
*HbMPK3*	scaffold0029 (+): 1022016–1025825	1119	373	42.62	7.62	Nuclear
*HbMPK4*	scaffold0074 (−): 2291777–2294826	1116	372	42.65	4.81	Nuclear
*HbMPK5*	scaffold0103 (−): 1887233–1894046	1152	384	43.69	6.54	Nuclear
*HbMPK6*	scaffold0153 (+): 394393–398696	1119	373	42.60	7.21	Cytoplasmic
*HbMPK7*	scaffold0192 (−): 249176–254990	969	323	36.92	8.85	Cytoplasmic
*HbMPK8*	scaffold0305 (−): 247836–257468	1689	563	64.10	8.75	Nuclear
*HbMPK9*	scaffold0458 (−): 777705–784024	1689	563	64.12	8.74	Nuclear
*HbMPK10*	scaffold0564 (−): 493867–503681	1845	615	70.01	9.27	Nuclear
*HbMPK11*	scaffold0650 (−): 11111–22341	1221	407	46.09	5.37	Nuclear
*HbMPK12*	scaffold0698 (+): 129035–139254	1848	616	69.95	9.28	Nuclear
*HbMPK13*	scaffold0734 (−): 555200–561767	1113	371	42.72	6.28	Nuclear
*HbMPK14*	scaffold0821 (+): 442427–452780	1158	386	44.55	6.20	Nuclear
*HbMPK15*	scaffold0949 (+): 291882–299015	1689	563	64.10	8.75	Nuclear
*HbMPK16*	scaffold1232 (−): 22626–37629	1230	410	46.45	5.45	Nuclear
*HbMPK17*	scaffold1373 (−): 131475–135479	894	298	34.85	5.89	Nuclear
*HbMPK18*	scaffold2000 (−): 35013–49042	1791	597	67.66	6.62	Nuclear
*HbMPK19*	scaffold2026 (−): 13507–24355	1221	407	46.09	5.37	Nuclear
*HbMPK20*	scaffold3133 (−): 1953–14125	1833	611	69.26	8.84	Nuclear

^a^ scaffold location: [scaffold number (orientation): start–end], ‘+’ and ‘−’ indicate forward and reverse orientations, respectively; ^b^ ORF, open reading frame; ^c^ aa, amino acid; MW, molecular weight; *p*I, isoelectric point; ^d^ the subcellular location of each HbMPK was predicted using the Softberry server.

**Table 2:** Identification and quantitative information of four validated HbMPKs.

HbMPK	Peptide Numbers	H_2_O-48 h (114)	Eth-48 h (115)	H_2_O-96 h (116)	Eth-96 h (117)
HbMPK12	2	1.0000 *	1.0715	0.8637	1.2319
HbMPK14	5	1.0000	2.5744	1.0183	2.3829
HbMPK16	4	1.0000	2.0222	1.0183	2.0445
HbMPK19	5	1.0000	1.9940	1.4755	1.9520

* Relative protein expression levels are defined as the ratio of 114/114, 115/114, 116/114 and 117/114. Eth, ethylene.

**Table 3:** The *Ka*/*Ks* ratios of duplicated *MPK* genes in *Hevea*.

Paralogous Genes	*Ka*	*Ks*	*Ka*/*Ks*	Selective Pressure
*HbMAPK11/HbMPK19*–*HbMAPK16*	0.0182	0.2533	0.0719	Purifying selection
*HbMAPK3*–*HbMAPK6*	0.0192	0.2068	0.0928	Purifying selection
*HbMAPK10*–*HbMAPK12*	0.0174	0.2326	0.0748	Purifying selection
*HbMAPK8/HbMPK15*–*HbMAPK9*	0.0034	0.1376	0.0247	Purifying selection

*Ka*, nonsynonymous substitution rate; *Ks*, synonymous substitution rate.

**Table 4:** Tajima relative rate tests of MPK gene pairs in rubber tree. ^a^

Testing Group	Mt ^b^	M1 ^c^	M2 ^d^	χ^2^	*p* ^e^
*HbMPK3/HbMPK6*–*MeMPK2*	348	6	11	1.47	0.22525
*HbMPK10/HbMPK12*–*MeMPK20*	513	16	36	7.69	0.00555
*HbMPK11/HbMPK16*–*MeMPK6*	369	10	16	1.38	0.23932
*HbMPK16/HbMPK19*–*MeMPK6*	369	10	16	1.38	0.23932
*HbMPK8/HbMPK9*–*MeMPK16-2*	520	4	5	0.11	0.73888
*HbMPK15/HbMPK9*–*MeMPK16-2*	520	4	5	0.11	0.73888

^a^ The Tajima relative rate test was used to examine the equality of the evolutionary rate between rubber tree paralogues; ^b^ Mt is the sum of the identical sites in all three sequences tested; ^c^ M1 is the number of unique differences in the first paralogue; ^d^ M2 is the number of unique differences in the second paralogue; ^e^ If *p* < 0.05, the test rejects the equal substitution rates between the two duplicates and infers that one of the two duplicates has an accelerated evolutionary rate.

## Supplementary Materials

The following are available online at www.mdpi.com/2073-4425/8/10/261/s1. Table S1: Primers used for PCR. Table S2: Putative *cis*-element sequences in the promoter regions of *HbMPK* genes. Figure S1: Raw spectra of peptides mapped to *HbMPK* proteins. Figure S2: Phylogenetic and exon-intron structure analyses of *MPK* genes in *M. esculenta* (*Me*) and *Hevea* (*Hb*).
