# Supplementary material for: Expression Profiling of Mitogen-Activated Protein Kinase Genes Reveals Their Evolutionary and Functional Diversity in Different Rubber Tree (Hevea brasiliensis) Cultivars
Source: Genes (Basel). 2017 Oct 6;8(10):261. doi: 10.3390/genes8100261 (PMC5664111; doi:10.3390/genes8100261)

**Supplementary Figure S1**. Raw spectra of peptides mapped to HbMPK proteins. The raw MS/MS spectra mapped to HbMPK proteins listed in Table 1 were shown. The m/z values were indicated. The amino acids of identified peptides were also indicated at the top of each spectrum.

MS/MS spectrum mapped to HbMPK12


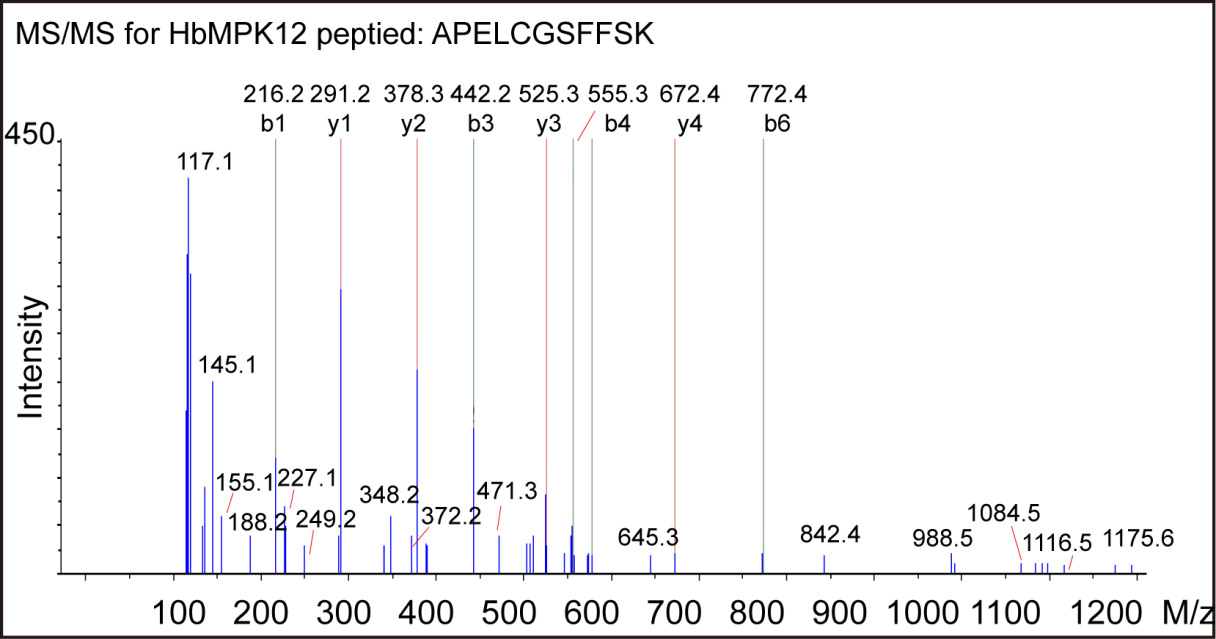


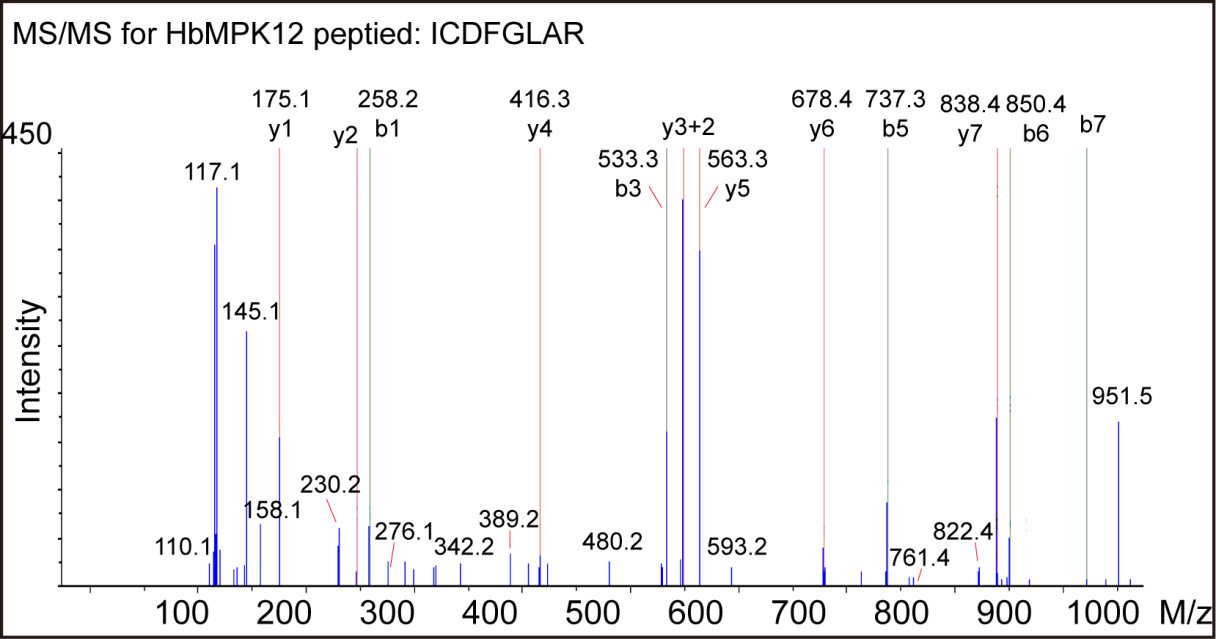


MS/MS spectrum mapped to HbMPK14


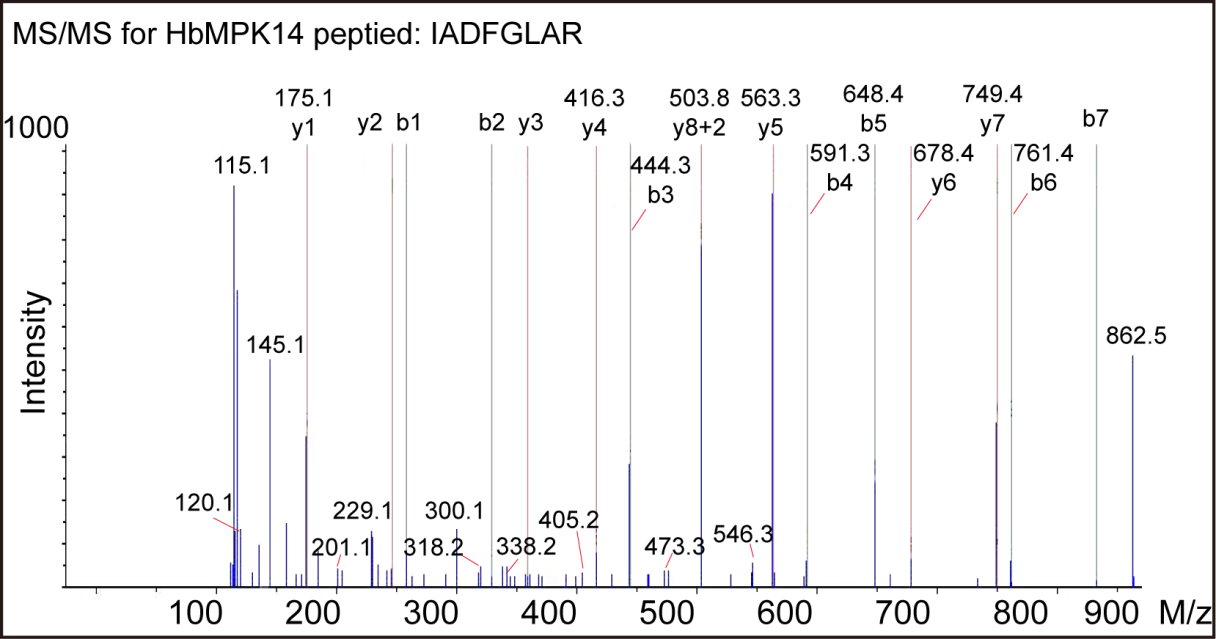


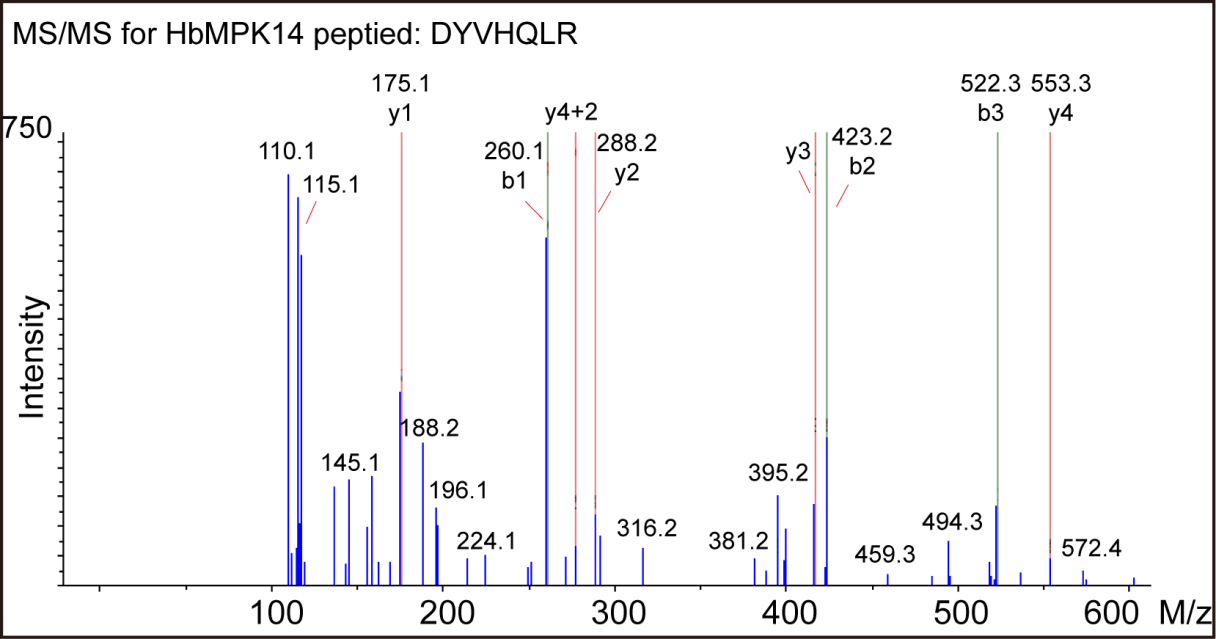


MS/MS spectrum mapped to HbMPK14


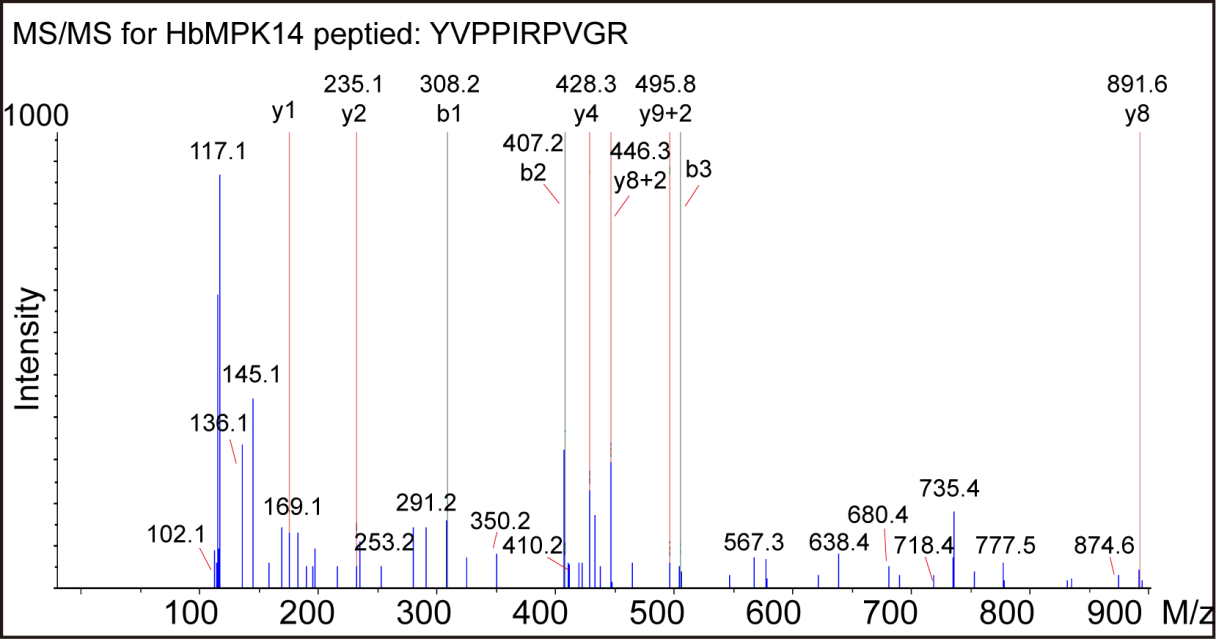


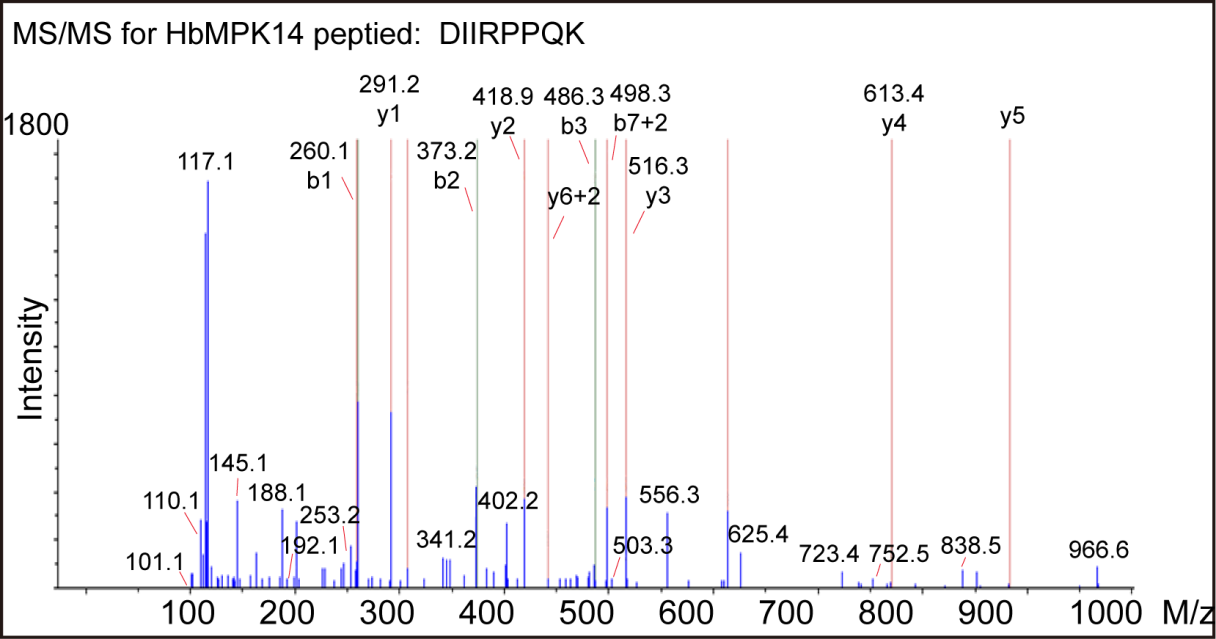


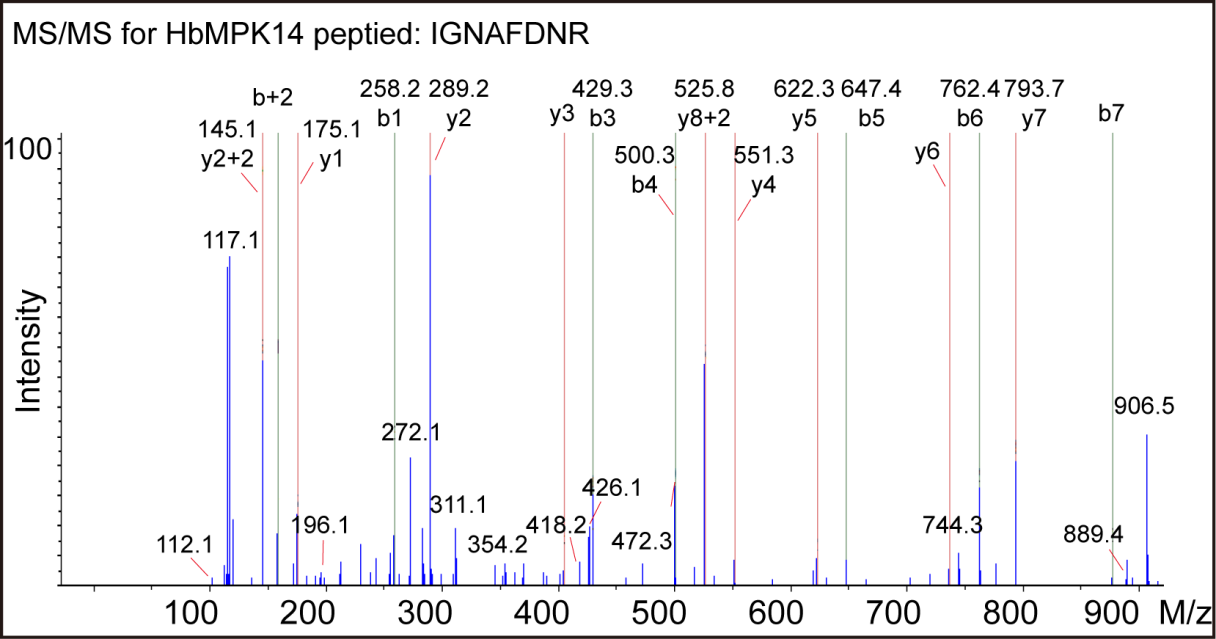


MS/MS spectrum mapped to HbMPK16


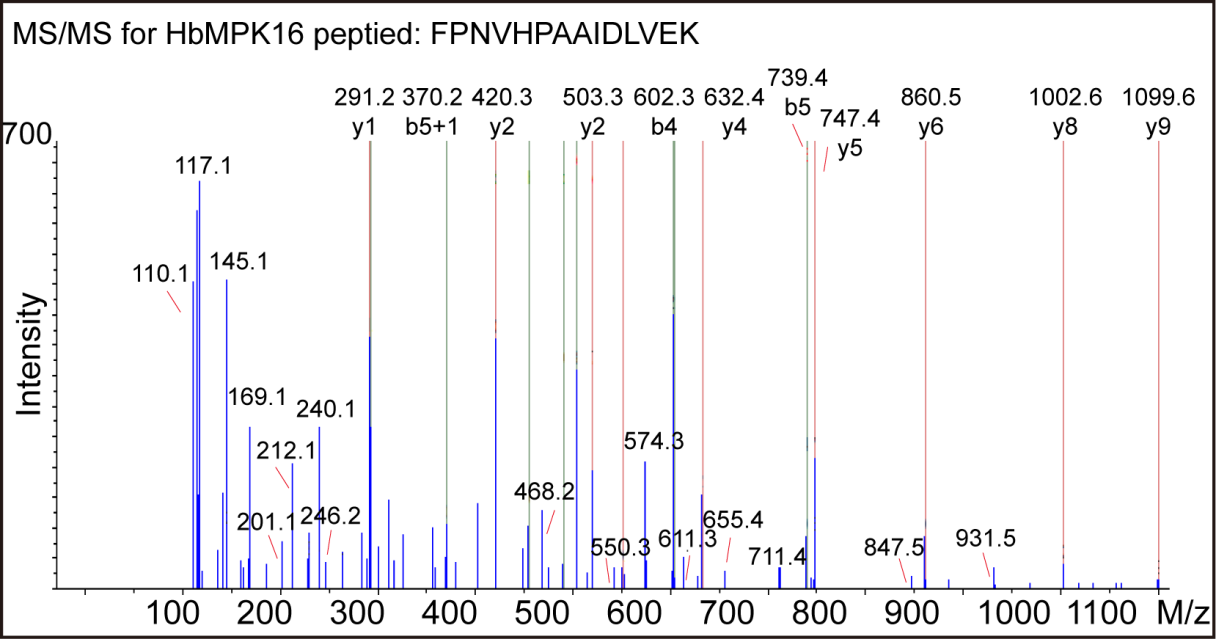


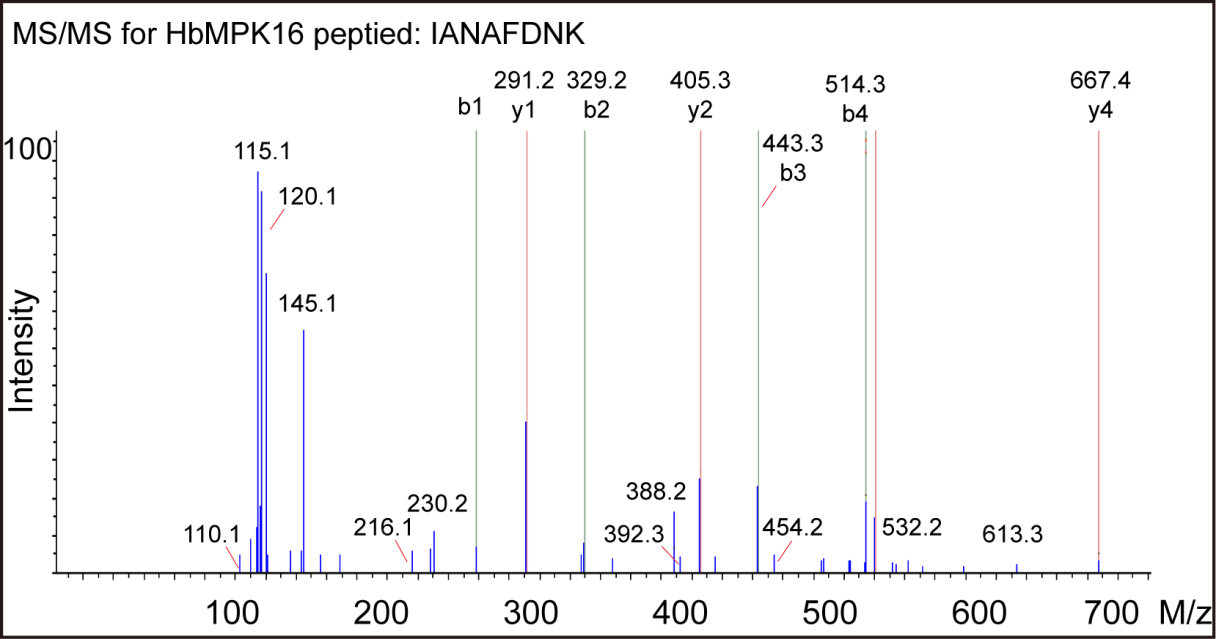


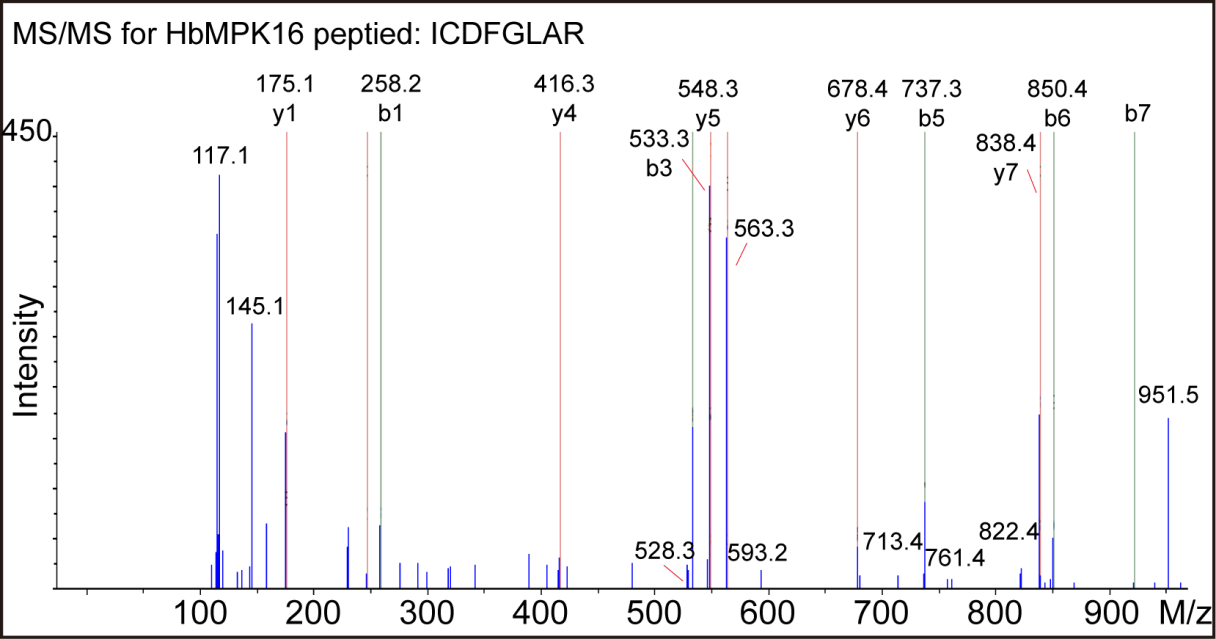


MS/MS spectrum mapped to HbMPK16


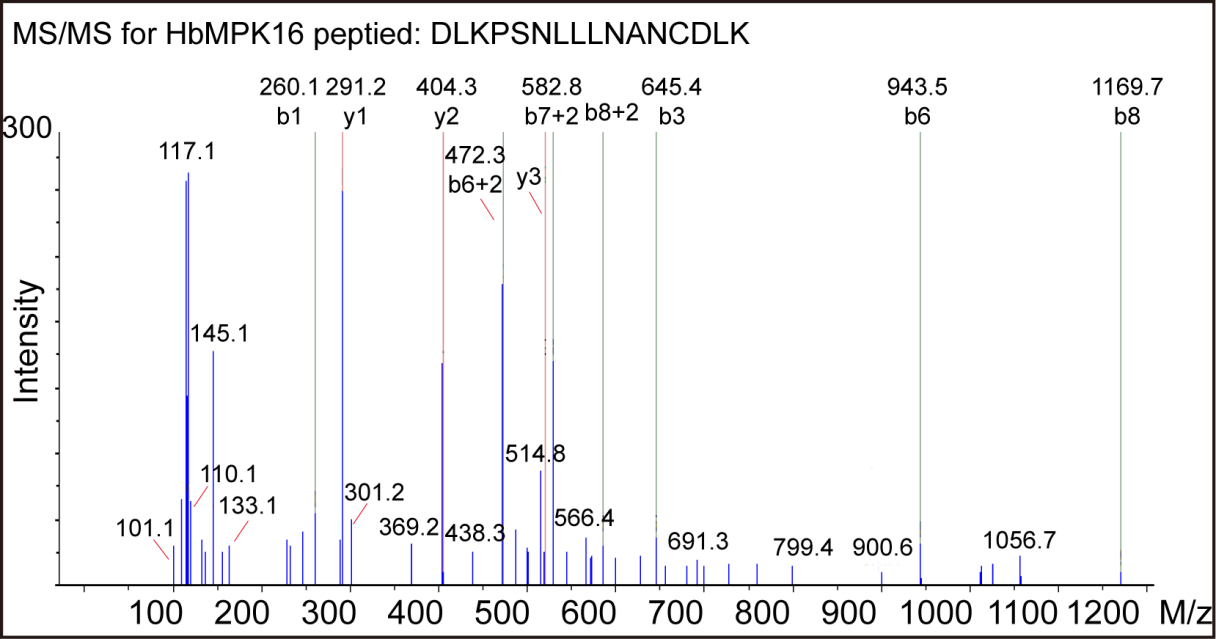


MS/MS spectrum mapped to HbMPK19


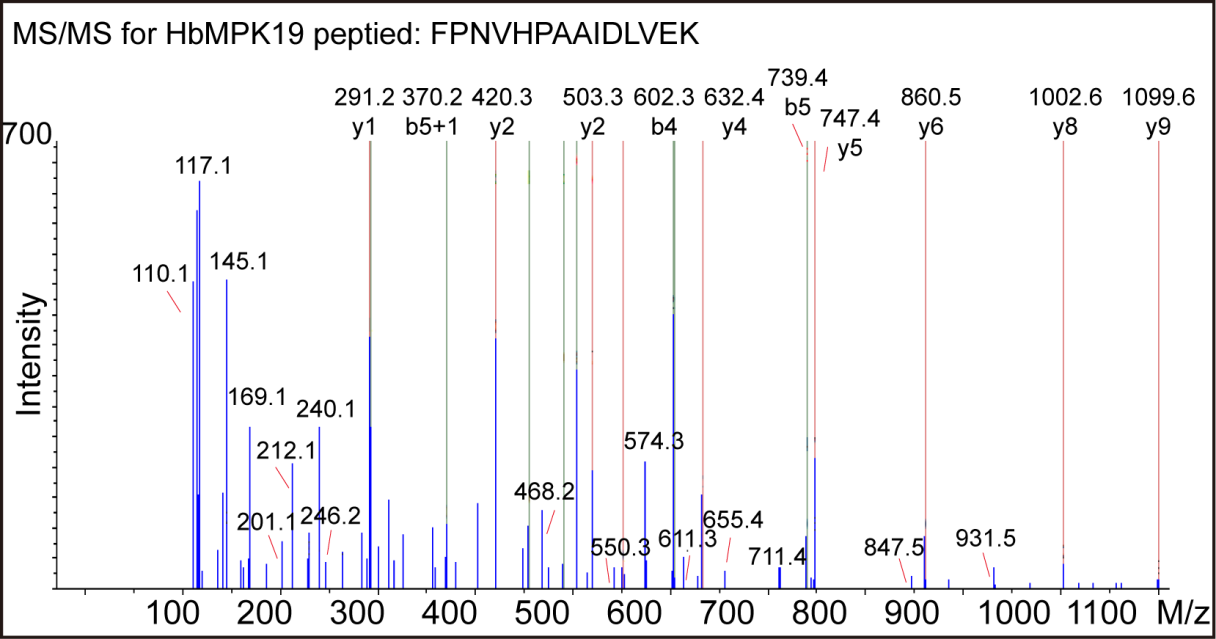


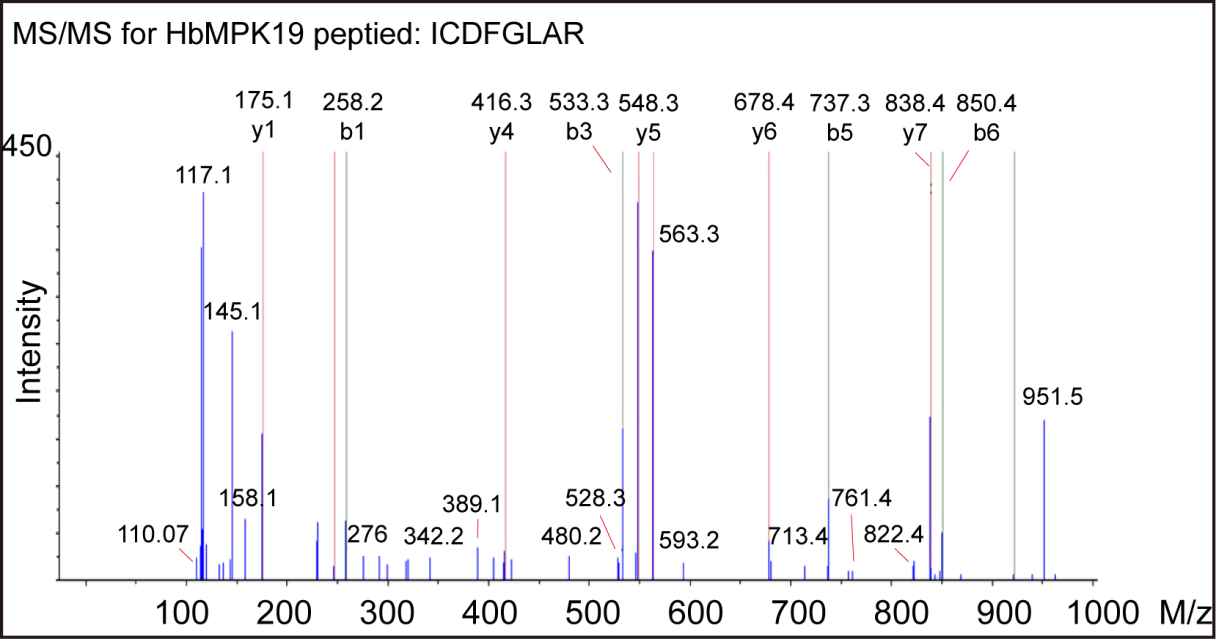


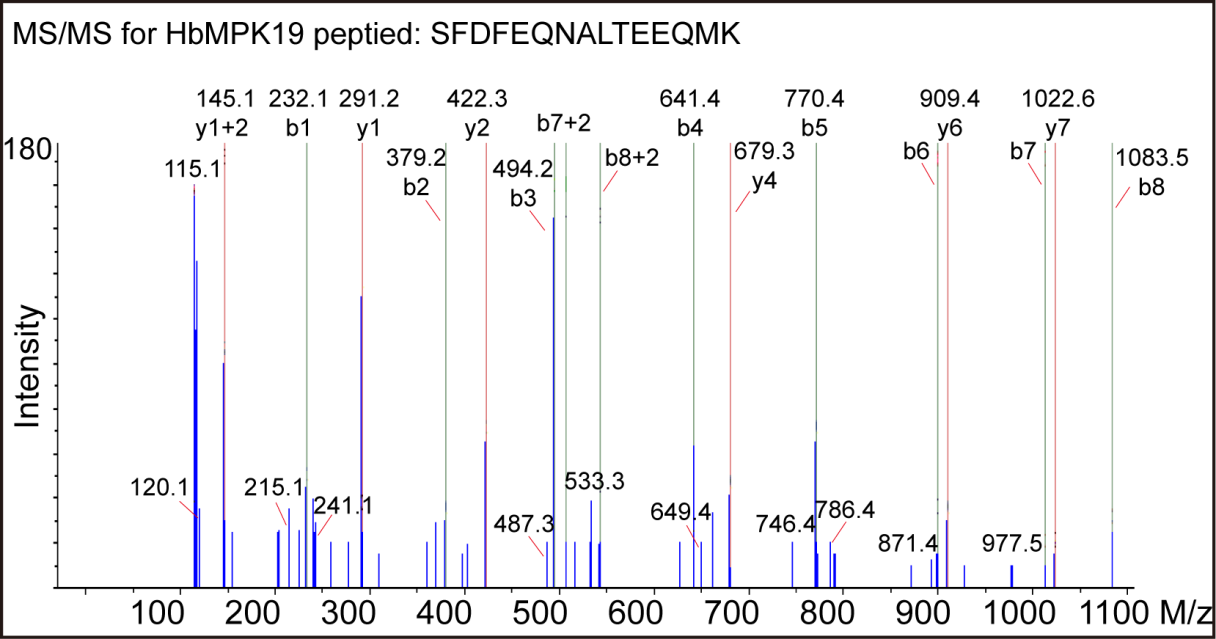


MS/MS spectrum mapped to HbMPK19


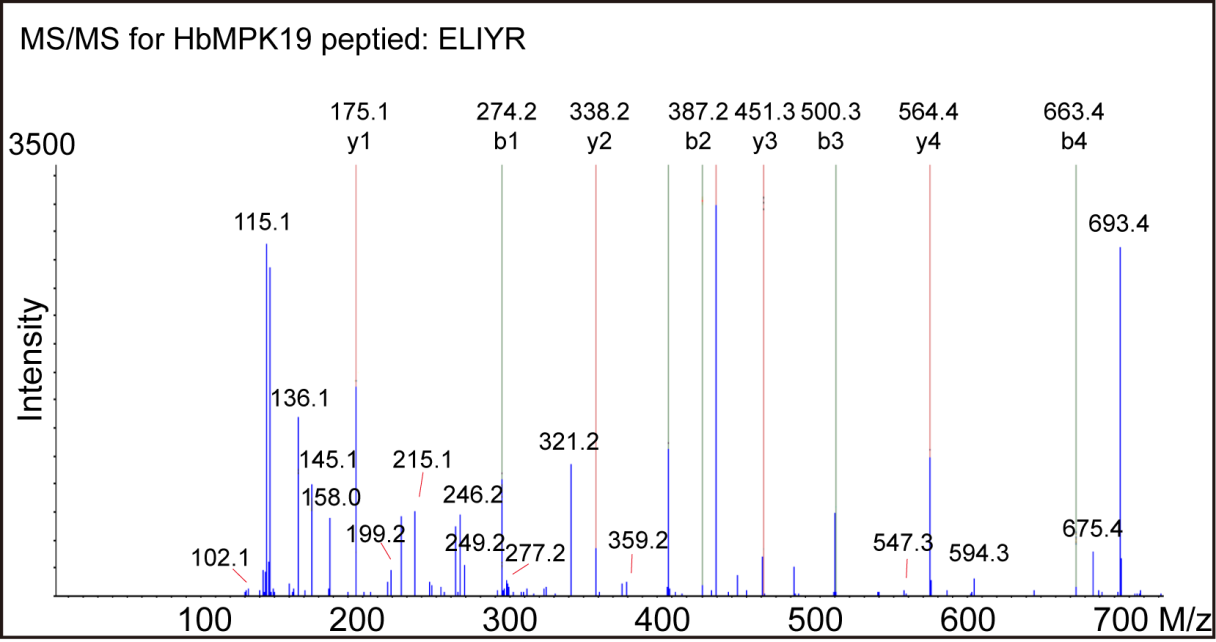


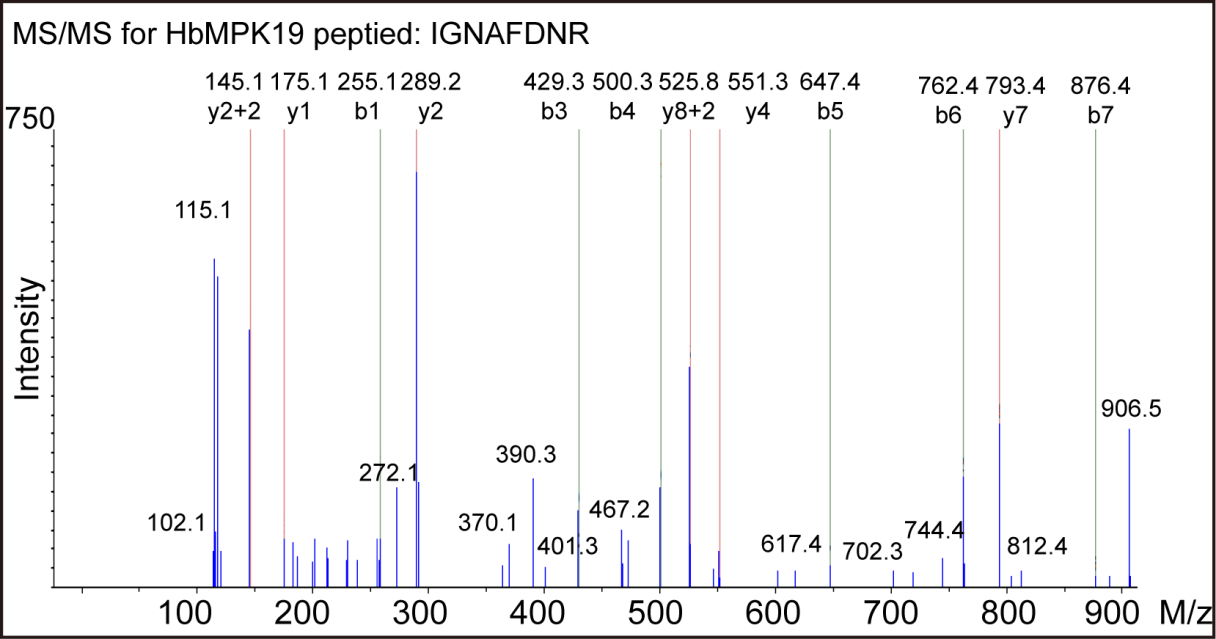


**Supplementary Figure S2**. Phylogenetic and exon-intron structure analyses of *MPK* genes in *M. esculenta* (Me) and *H. brasiliensis* (Hb). The phylogenetic tree (left) was constructed using MEGA5.0 as described above. Exon-intron distribution was analyzed using GSDS software (right). Introns and exons were represented by lines and yellow boxes. The scale bar for genomic length was indicated at the bottom.


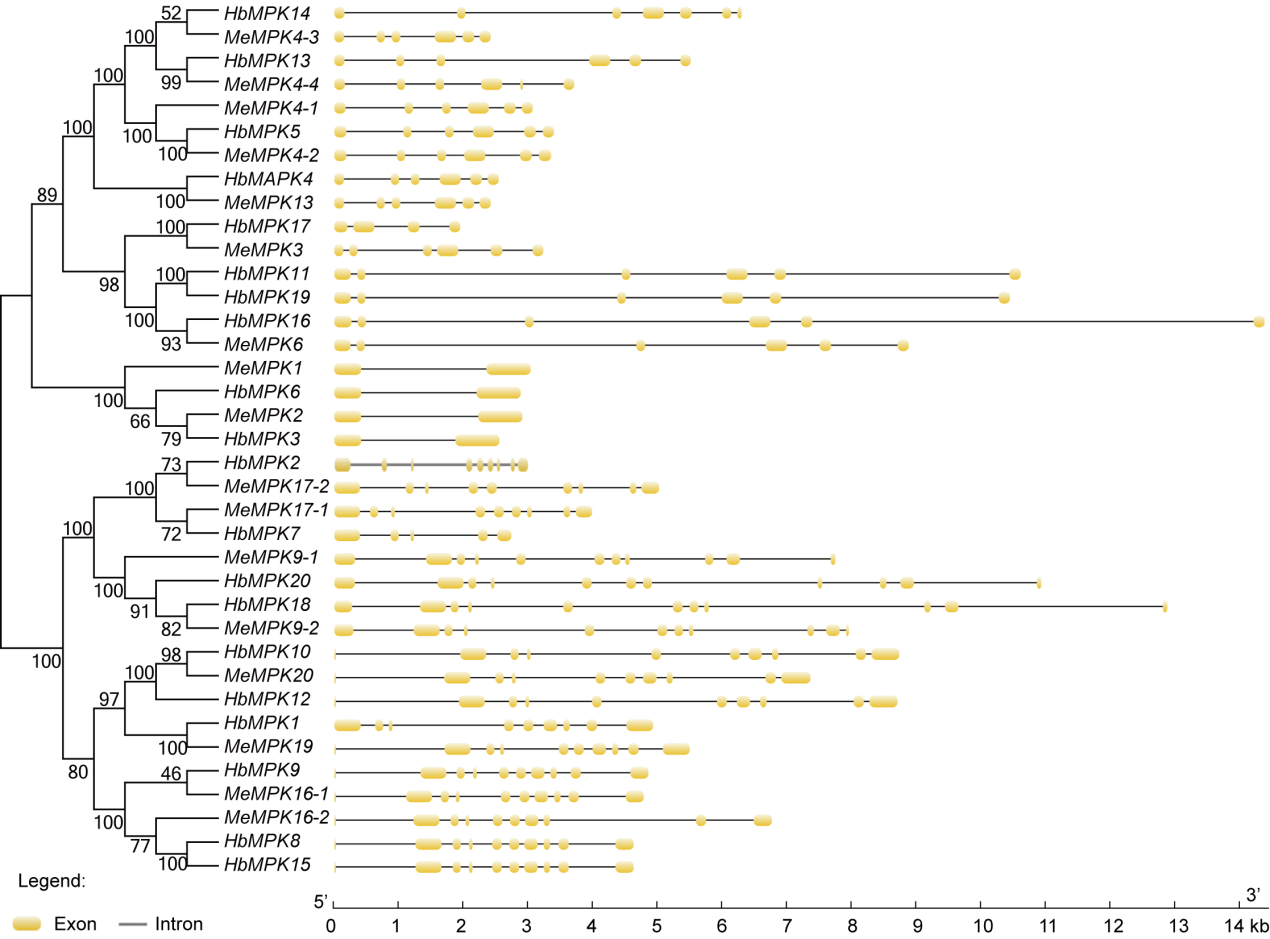

Supplement: Supplementary file 1 [file genes-08-00261-s001.zip › Supp data/Supp_Figures.docx]
