# Supplementary material for: Expression Profiling of Mitogen-Activated Protein Kinase Genes Reveals Their Evolutionary and Functional Diversity in Different Rubber Tree (Hevea brasiliensis) Cultivars
Source: Genes (Basel). 2017 Oct 6;8(10):261. doi: 10.3390/genes8100261 (PMC5664111; doi:10.3390/genes8100261)
Supplement: Supplementary file 1 [file genes-08-00261-s001.zip › Supp data/Supp_Tables.docx]

**Table S1. Primers used in this work.**

| **Primer** | **name** | **Primer sequence (5'-3')** | **Production size (bp)** |
| --- | --- | --- | --- |
| *HbMPK1* | sense | GAGAGACGAAGGGTGACAAAGG | 197 |
|  | anti-sense | GGAATCACTGGTGCACTTCGAC |  |
| *HbMPK2* | sense | GAAAAAACGACCAATTCCTTTG | 158 |
|  | anti-sense | GCTCCTTATTTGCCAGATCATG |  |
| *HbMPK3* | sense | GATACGAAATATGTGCCCATAAAA | 266 |
|  | anti-sense | TCCATGAGTTCATACACCAGATAG |  |
| *HbMPK4* | sense | CCAGATGTGTCAGCAGTGGCTC | 149 |
|  | anti-sense | AAAGGAGATGGGCAAGTAGGCT |  |
| *HbMPK5* | sense | TACTGGGGCTGCTGATCTGC | 203 |
|  | anti-sense | AATCCCTCCAAATGAGTTCCTT |  |
| *HbMPK6* | sense | AGGGGGAGGTGGATGTCG | 304 |
|  | anti-sense | CTCCACATCGTCTCCCGTATC |  |
| *HbMPK7* | sense | GATTTGCTTGGCACTCCTTC | 163 |
|  | anti-sense | ATGCCAGTAAACGCTCAAGTAA |  |
| *HbMPK8/15* | sense | GAACAACGACGGGTGGTCAG | 145 |
|  | anti-sense | GCTTTGGCTGCAATCCATTA |  |
| *HbMPK9* | sense | ATTTAATGATACCCCAACTGCG | 167 |
|  | anti-sense | GGAAAAGAGGTTTTCCTGTCAA |  |
| *HbMPK10* | sense | GGGCAGGAATGAATAAGGTCGA | 112 |
|  | anti-sense | CTGTGAGAAGCCGGGGCA |  |
| *HbMPK11/19* | sense | CGCTCATCCCTACCTAACATCAC | 109 |
|  | anti-sense | TCATCTGCTCTTCGGTCAATG |  |
| *HbMPK12* | sense | TGCCTATGACCCAAGAACTTTTGTC | 240 |
|  | anti-sense | TCCACCCTATTCACTCCTGCCC |  |
| *HbMPK13* | sense | ACAGAGCTCATTGGTTCACCG | 301 |
|  | anti-sense | CCTCGGTAAATGTTGGCTGC |  |
| *HbMPK14* | sense | TGATGAGGCACTGTGCCACC | 182 |
|  | anti-sense | CGAGAGAGTTCTTTTCTTCTTTCTG |  |
| *HbMPK16* | sense | AGAGGCTGAGTTGGGGTTTTTG | 290 |
|  | anti-sense | GCTCCTTCATCAGTTCTTCAGTCATC |  |
| *HbMPK17* | sense | ATCCGACAACTAAGTCAATATCCTC | 248 |
|  | anti-sense | ATTTCCTTCATCTGCTCTTCTACCA |  |
| *HbMPK18* | sense | CAACCCAGTCCTAAGGCACCAT | 192 |
|  | anti-sense | TTCAATAAGCTACGAGCAGAGTCA |  |
| *HbMPK20* | sense | TCATCGAGACTTAAAGCCCAAA | 284 |
|  | anti-sense | TGAGATCCAATTGATGCACTACA |  |
| *HbActin* | sense | CAGGGCAGTGTTTCCCAGTATAG | 379 |
|  | anti-sense | CAGCACGATACCAGTTGTACGAC |  |

**Table S2. Putative *cis*-element sequences in promoter regions of *HbMPK* genes.**

| **Classification** | **Symbol** | ***cis*-element** | **Sequence *** | **Description** |
| --- | --- | --- | --- | --- |
|  |  |  |  |  |
| Transcription initiation | T | TATA box | TATAAAT | Core *cis*-element influenced the efficiency of transcription in eucaryote |
|  | C | CAAT box | CCAAT | Common *cis*-element in eukaryotic promoter and enhancer regions |
| Phytohormone responsiveness | A | ABRE | TACGTG | Abscisic acid (ABA)-responsive element |
|  | E | ERE | AWTTCAAA | Ethylene-responsive element |
|  | G | GARE | WAACAR | Gibberellin-responsive element |
|  | J | TGACG-motif | TGACG | *cis*-acting regulatory element involved in the MeJA-responsiveness |
|  | F | TCA-element | CCATCTTTTT | *cis*-acting element involved in salicylic acid responsiveness |
|  | P | P-box | CCTTTTG | Gibberellin-responsive element |
| Stress responsiveness | M | MBS | YAACTG | MYB binding site involved in drought-inducibility |
|  | H | HSE | CTNGAANNTTCNAG | Heat shock-responsive element |
|  | K | TC-rich repeats | RTTTTCTYMM | *cis*-element involved in defense and stress responsiveness |
|  | L | G-box | YACGTN | *cis*-regulatory element involved in light responsiveness |
|  | R | ARE | TGGTTT | *cis*-acting regulatory element essential for the anaerobic induction |
|  | Q | AE-box | AGAAACTT | part of a module for light response |
|  | W | box-W1 | TTGACC | fungal elicitor responsive element |
|  | S | Skn-1_motif | GTCAT | *cis*-acting regulatory element required for endosperm expression |
|  | B | Box I | TTTCAAA | light responsive element |
|  | O | LTR | CCGAAA | *cis*-acting element involved in low-temperature responsiveness |
|  | N | WUN-motif | TCATTACGAA | Wound-responsive element |
| High transcription | U | 5`UTR Py-rich stretch | TTTCTTCTCT | *cis*-regulatory element conferring high transcription levels |

* N = A, C, G or T; M = A or C; R = A or G; W = A or T; Y = C or T.
